# Two Novel Mutations on Exon 8 and Intron 65 of *COL7A1* Gene in Two Chinese Brothers Result in Recessive Dystrophic Epidermolysis Bullosa

**DOI:** 10.1371/journal.pone.0050579

**Published:** 2012-11-30

**Authors:** Ying Lin, Xue-Jun Chen, Wei Liu, Bo Gong, Jun Xie, Jun-Hao Xiong, Jing Cheng, Xi-Ling Duan, Zhao-Chun Lin, Lu-Lin Huang, Hui-Ying Wan, Xiao-Qi Liu, Lin-Hong Song, Zheng-Lin Yang

**Affiliations:** 1 The Sichuan Provincial Key Laboratory for Human Disease Gene Study, Sichuan Academy of Medical Sciences & Sichuan Provincial People’s Hospital, No.32, Western 2nd Section, Chengdu, Sichuan, P. R. China; 2 Institute of Dermatology and Venereology, Sichuan Academy of Medical Sciences & Sichuan Provincial People’s Hospital, No. 32, Western 2nd Section, Chengdu, Sichuan, P. R. China; 3 Department of Pathology, Sichuan Academy of Medical Sciences & Sichuan Provincial People’s Hospital, No. 32, Western 2nd Section, Chengdu, Sichuan, P. R. China; Ohio State University Medical Center, United States of America

## Abstract

Dystrophic epidermolysis bullosa is an inherited bullous dermatosis caused by the *COL7A1* gene mutation in autosomal dominant or recessive mode. *COL7A1* gene encodes type VII collagen – the main component of the anchoring fibrils at the dermal–epidermal junction. Besides the 730 mutations reported, we identified two novel *COL7A1* gene mutations in a Chinese family, which caused recessive dystrophic epidermolysis bullosa (RDEB). The diagnosis was established histopathologically and ultrastructurally. After genomic DNA extraction from the peripheral blood sample of all subjects (5 pedigree members and 136 unrelated control individuals), *COL7A1* gene screening was performed by polymerase chain reaction amplification and direct DNA sequencing of the whole coding exons and flanking intronic regions. Genetic analysis of the *COL7A1* gene in affected individuals revealed compound heterozygotes with identical novel mutations. The maternal mutation is a 2-bp deletion at exon 8 (c.1006_1007delCA), leading to a subsequent reading frame-shift and producing a premature termination codon located 48 amino acids downstream in exon 9 (p.Q336EfsX48), consequently resulting in the truncation of 2561 amino acids downstream. This was only present in two affected brothers, but not in the other unaffected family members. The paternal mutation is a 1-bp deletion occurring at the first base of intron 65 (c.IVS5568+1delG) that deductively changes the strongly conserved GT dinucleotide at the 5′ donor splice site, results in subsequent reading-through into intron 65, and creates a stop codon immediately following the amino acids encoded by exon 65 (GTAA→TAA). This is predicted to produce a truncated protein lacking of 1089 C-terminal amino acids downstream. The latter mutation was found in all family members except one of the two unaffected sisters. Both mutations were observed concurrently only in the two affected brothers. Neither mutation was discovered in 136 unrelated Chinese control individuals. This study reveals novel disease-causing mutations in the *COL7A1* gene.

## Introduction

Dystrophic epidermolysis bullosa (DEB) is an inherited bullous dermatosis characterized by high skin fragility. The phenotype of this disease is variable [1]. Common symptoms include skin blisters, scars, pseudosyndactyly, and onychodystrophy caused by slight trauma. In severe patients, mutilating scarring of the hands and feet, and squamous cell carcinoma may develop, possibly inducing death.

DEB is the consequence of *COL7A1* gene (MIM *120120) mutation in autosomal dominant (DDEB, MIM#131750) or recessive (RDEB, MIM#226600) fashion [2]. *COL7A1* gene encodes type VII collagen, the main component of the anchoring fibrils at the sub-lamina densa of the dermo-epidermal junction [3].

More than 730 mutations of *COL7A1* gene have been reported, demonstrating the high heterogeneity associated with the disease [4]. Here we report two novel mutations in the *COL7A1* gene resulting in RDEB in two brothers from a Chinese family.

## Materials and Methods

### Ethics Statement

All the analyses have been approved by the ethics committee of the Sichuan Academy of Medical Sciences & Sichuan Provincial People’s Hospital, and all clinical investigations have been conducted according to the principles expressed in the Declaration of Helsinki, with patients’ written informed consent.

### Patients and Clinic

**Figure 1:**
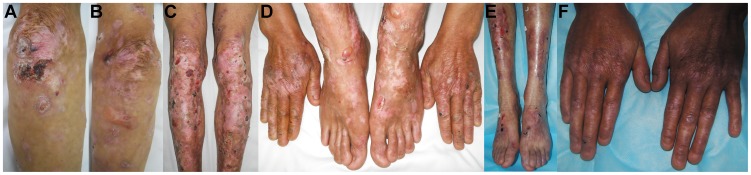
Clinical manifestations of the two RDEB patients. Blisters, blood blisters, scarring formation, and hypopigmentation on the dorsal side of the elbows (A, B), tibialis, knees (C), hands, and feet could be seen in the proband. Nails of fingers and toes were all absent (D). Similar lesions could be seen in the proband’s younger brother (E, F).

**Figure 2:**
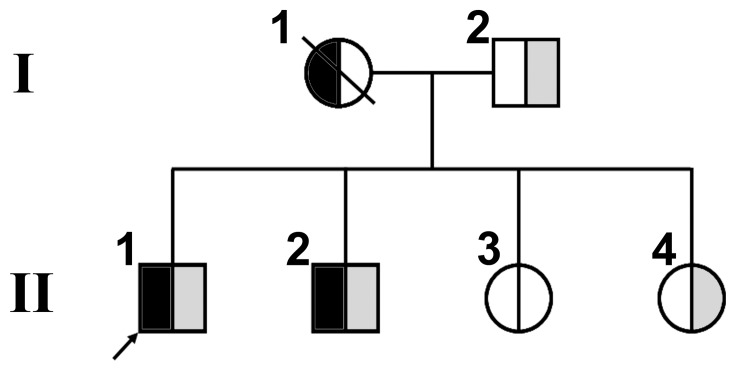
Pedigree of the RDEB family. The segregation of the mutations of c.1006_1007delCA on exon 8 (half black symbols) and c.IVS5568+1delG at the donor splicing site of intron 65 (half gray symbols) on the *COL7A1* gene is shown. The arrow refers to the proband.

The proband was a 42-year-old man suffering from blisters, blood blisters, and scarring formation primarily on the dorsal side of the hands, feet, elbows, tibialis, and knees after slight trauma since birth. Some hypopigmentation could also be seen in the lesion area. No lesions were found on the trunk or mucous membrane. The nails on his fingers and toes never grew (Fig. 1A, B, C, and D). There was no extracutaneous involvement. The proband’s 33-year-old younger brother (Fig. 1E, F) – but not his two sisters, his father, and all relatives – had the similar experience and lesions (Fig. 2). The proband’s parents were not consanguine. His mother, who died of leukemia, was reported to have no bullous dermatosis in her lifetime.

Biopsy of the proband was performed for histopathologic examination, direct immunofluorescence assay, and transmission electron microscopy. Blood routine and serum biochemical assay of the two patients, their unaffected father, and two sisters were also investigated.

### Mutation Screening

Genome DNA was extracted from the peripheral leucocytes from EDTA-anticoagulated peripheral blood samples of the two patients, their unaffected father, and two sisters, followed by polymerase chain reaction (PCR) with 72 pairs of primers in two patients. PCR primers were designed to include flanking intronic sequences of each exon according to published protocols [5]. Specifically, the two pairs of primer with which the mutations were found were: (L) 5′ CTTGGCTGGGCAAGATAAAG 3′ and (R) 5′CAGCCCACATCTCTCACTCA 3′ for exon 8, and (L) 5′ATGCAGGCACACCCTTAGAC 3′ and (R) 5′ GGGTCAGAGGTTGCAGAAAA 3′ for exon 65 and intron 65. For PCR amplification, 2.0 µl (25 ng/µl) of genome DNA was used as template in 20 µl of reaction mixture containing 1.6 µl dNTPs (2.5 mmol/L), 1.2 µl Mg^2+^ (25 mmol/L), 1 µl of each primer (10 µmol/L), and 0.4 µl AmpliTaq Gold polymerase (5 U/µl). PCR conditions were 95°C for 5 min followed by 95°C for 30 s, 57–60°C for 30 s, and 72°C for 1 min (35 cycles), and finally extended at 72°C for 10 min. The PCR products, examined by 2% agarose gel electrophoresis, were 362 bp (for exon 8) and 410 bp (for exon 65) in size, respectively.

The PCR products were purified by using QIA quick Gel Extraction Kit (QIAGEN, Valencia, CA, USA), and sequenced by BigDye Terminator Cycle Sequencing Ready Reaction Kit (Applied Biosystems) on ABI 3100 Genetic Analyzer (Applied Biosystems). The mutation locations were determined by BLAST comparing the results with the original sequence in GenBank.

### Mutation Verification

To verify the novel mutations found in the two patients, genome DNA of their remaining three family members and 136 normal controls unrelated to this family and disease were also amplified with primers for exon 8 and exon 65 and sequenced.

## Results

### Clinical Findings

**Figure 3:**
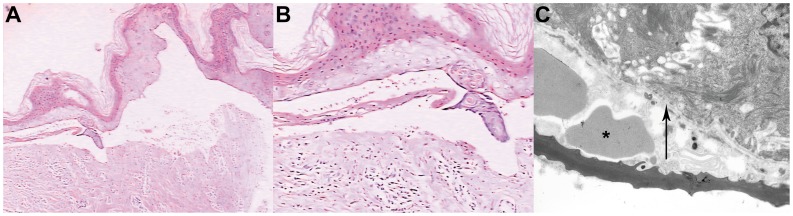
Histopathologic and ultrastructural features of the RDEB proband’s skin lesion. Hematoxylin and eosin stain (H&E) of the skin lesion indicated a keratinocytes degeneration and necrosis, subepidermal bulla with infiltration of a few inflammatory cells (A, magnification×40; B×100). Under transmission electron microscope, the split was located at the sub-lamina densa where some red blood cells could be seen (asterisk) (C). The arrow refers to the lamina densa. Magnification×10000.

Histopathologic examination of the proband indicated a keratinocyte degeneration and necrosis. subepidermal bulla with infiltration of a few inflammatory cells (Fig. 3A, B). Direct immunofluorescence assay showed negative stain of IgG, IgA, IgM, complement C3, and C4 at the dermo-epidermal junction (data not shown). Under transmission electron microscope, the split was located at the sub-lamina densa where some red blood cells could be seen (Fig. 3C). The diagnosis of RDEB (generalized, other type) was made according to Fine JD et al. [6]. The results of blood routine and serum biochemistry examination of the two patients, their two sisters, and father were all within the normal range.

### Sequencing Analysis

**Figure 4:**
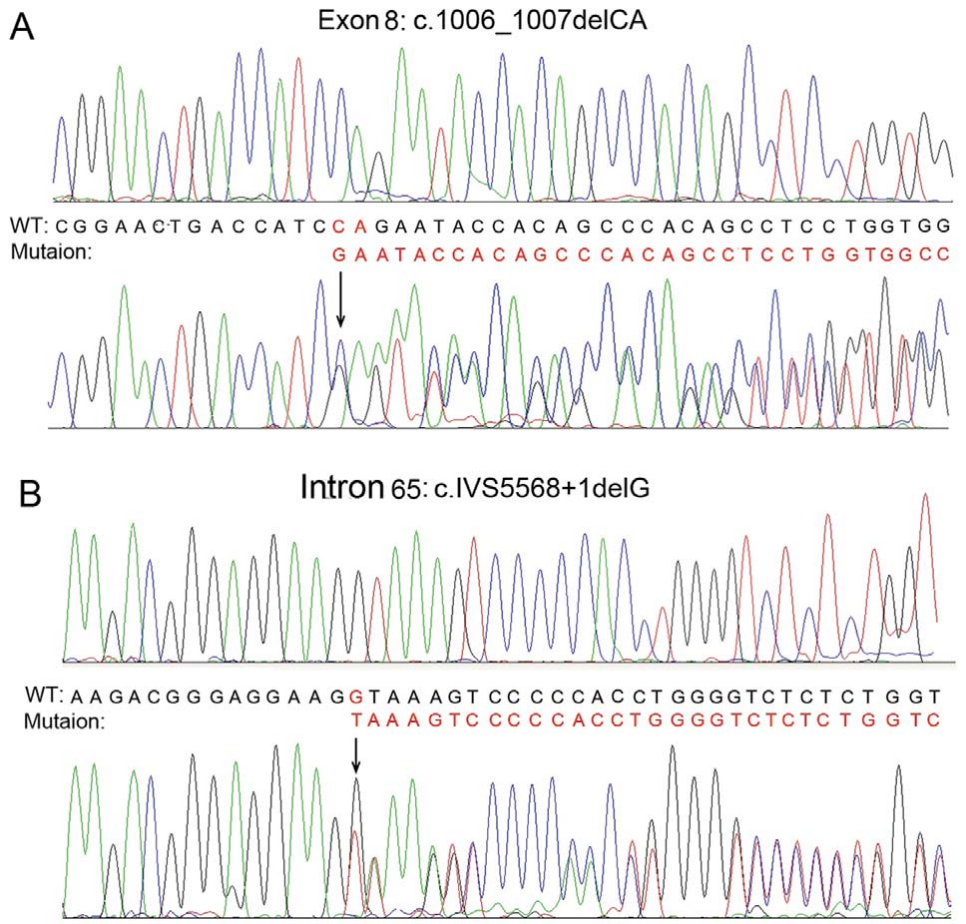
Result of DNA sequencing of *COL7A1* mutation in the affected brothers. It shows compound heterozygosity of the mutations of c.1006_1007delCA (exon 8) (A) and c.IVS5568+1delG (intron 65) (B).

DNA sequencing of exons and flanking introns of the two patients disclosed that both the brothers were compound heterozygotes with identical mutations. One of the two mutations found in the two patients, but not their father and two sisters, was located at exon 8 with c.1006_1007delCA (Fig. 4A). The other novel mutation in the other chain of the alleles in the two patients, their younger sister, and father was c.IVS5568+1delG located at position -1 of the donor splicing site of intron 65 (Fig. 4B). Both the two mutations coexisted simultaneously only in the two brothers. None of the 136 controls unrelated to this family, without bullous dermatosis, was found to be the carrier of either of the two mutations.

## Discussion

DEB may be inherited in autosomal dominant or autosomal recessive pattern, both of which are induced by *COL7A1* gene mutation. DDEB and RDEB are divided into 6 and 7 subtypes, respectively [6]. Our study focuses on the latter. Comparatively speaking, the clinical phenotype of RDEB is more serious than that of DDEB.

**Figure 5:**
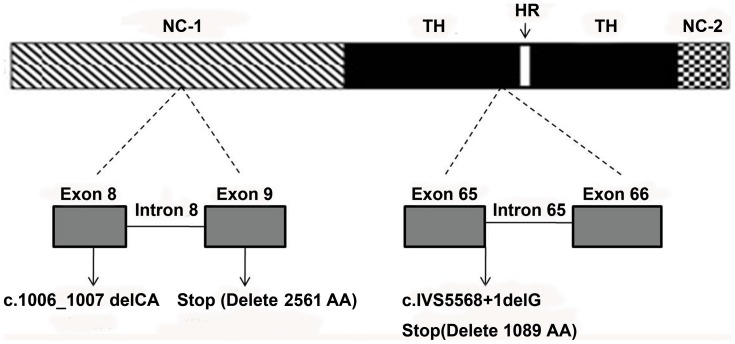
Schematic diagram of the consequences of the mutations identified in the RDEB pedigree. The mutation on exon 8 (c.1006_1007delCA), standing at NC-1 of the *COL7A1* gene, leads to a premature termination codon (stop) located 48 amino acids downstream in exon 9, resulting in truncation of 2561 amino acids downstream, which include part of the NC-1, all the collagenous triple helix, and the NC-2. The mutation discovered on intron 65 (c.IVS5568+1delG), located at the collagenous triple helix domain of the *COL7A1* gene, immediately creates a stop codon, consequently producing a truncated protein lacking part of 1089 C-terminal amino acids downstream. Abbreviations: TH, triple helix; HR, hinge region; NC-1, amino terminus; NC-2, carboxyl terminus.

*COL7A1* gene is located in chromosome 3p21.31. It is primarily expressed in stratified squamous epithelia, such as the skin, the mucous membranes, and the cornea of the eye [7]. Among all genes described so far, *COL7A1* gene is the second largest, containing 118 exons [5], [7], [8]. Its 8.8 Kb coding region of the 9.2 Kb transcript encodes pro-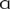
1 collagen (320 kDa) with distinct structural domains. This collagen, namely the type VII collagen, is composed of 2294 amino acids. The main structure of the molecule consists of a collagenous triple helix (145 kDa) encoded by exons 29–112, and an amino terminus (NC-1) (145 kDa) encoded by exons 2–28, as well as a carboxyl terminus (NC-2) encoded by exons 113–118 (30 kDa) [9]. The protein encoded by *COL7A1* gene is essential for the connection of the dermis and the epidermis.

The mutation of the *COL7A1* gene may result in DEB, in either autosomal dominant or autosomal recessive fashion. So far, more than 730 mutations on the *COL7A1* gene have been revealed to be involved in DEB, most of which are glycine substitution mutations located at the triple helix domain [10]. It has been reported that approximately 75% of the DDEB mutations occur in exons 73–75 [11], while RDEB are caused by mutations on both alleles that result in either null alleles or out-of-frame mutations from insertions/deletions, single-base substitutions, and splice junction alterations [12]–[16]. Known RDEB-induced mutation loci are scattered throughout the genome of the *COL7A1* gene, with no obvious hot spot [17]–[28]. Similar circumstances also exist in Chinese RDEB patients [29]–[31]. Although most RDEB were induced by compound heterozygous mutations, the majority of severe mutilating RDEB were caused by homozygous mutations leading to premature translational termination [32].

In this study, the maternal mutation is a 2-bp deletion at exon 8 of the *COL7A1* gene (c.1006_1007delCA), leading to a subsequent reading frame-shift and substitution of the codon of CAG, which encodes glutamine, by GAA, which encodes glutamic acid (p.Q336EfsX48). This accordingly generates a premature termination codon at 48 amino acids downstream of exon 9, resulting in the truncation of 2561 amino acids downstream, which include part of the NC-1 (from fibronectin 2 domain), all the collagenous triple helix, and the NC-2 (Fig. 5). As a consequence, the type VII collagen chain of the amino-terminus that functions as the adhesion site to the laminin 5 at the lamina densa, became defective, thus markedly affecting the adhesive function of the anchoring fibrils.

The paternal mutation is a 1-bp deletion at the first basepair of intron 65 (GTAA→TAA), resulting in subsequent read-through intron 65 and immediately creating a stop codon after the amino acids encoded by exon 65 (c.IVS5568+1delG). Due to inactivation of the strongly conserved GT dinucleotide at the 5′ donor splice site, it is predicted to produce a truncated protein lacking part of 1089 C-terminal amino acids downstream (Fig. 5).

As the carriers of either mutation, the patients’ younger sister and their father did not show any phenotype. Considering the fact that the two brothers with compound heterozygosis showed clinical RDEB, and their father was the carrier of the mutation c.IVS5568+1delG, we could presume that the patients’ asymptomatic mother should be the carrier of the mutation c.1006_1007delCA, although we could not detect it since she was deceased (Fig. 2).

The severity of RDEB may be related to the position of the stop codon; however, the presence of some functional protein may also appears to be the important factor in ameliorating the disease severity. Mutations occurring within the central collagenous domain allow preservation of the noncollagenous extensions, which can serve adhesive functions. Furthermore, it has been reported that the heterozygous carriers, which contain only about half the normal amount of anchoring fibrils in their skin, are clinically normal [33]. This may be part of the reason that the carriers of c.1006_1007delCA mutation (patients’ mother) or c.IVS5568+1delG mutation (patients’ father and younger sister) did not demonstrate any phenotype. Also, it has been clarified that the same mutation on *COL7A1* gene may result in a different phenotype of either dominant or recessive DEB [16], [34], [35], strongly suggesting that not only the mutation location, but also the nature of the mutation functionally affects helix formation, protein folding, thermal stability, intracellular transport, secretion, and assembly into anti-parallel dimmers or anchoring fibrils [18], [36], [37].

Neither mutation existed in 136 normal controls unrelated to this family, indicating that the mutations found in the two patients were not genetic polymorphism. Identification of two novel mutations in the human *COL7A1* gene is based on comparisons with the Human Genome Mutation Database. Despite the high heterogeneity in the *COL7A1* gene, the mutations found in this study are new mutations reported for the first time. Although over 730 mutations have been identified in the *COL7A1* gene, every novel mutation identified will provide a clue for early diagnosis of the disease in the future, and this compound mutation is the first mutation identified in the *COL7A1* gene in dystrophic epidermolysis bullosa in the western Chinese population. So the identification of this novel mutation is not only important for the family in the study, but also useful to study the pathogenesis of the disease in the future.
